# COVID-19 in Sammelunterkünften für Geflüchtete: Analyse von Pandemiemaßnahmen und prioritäre Bedarfe aus behördlicher Sicht

**DOI:** 10.1007/s00103-021-03284-2

**Published:** 2021-02-09

**Authors:** Louise Biddle, Rosa Jahn, Clara Perplies, Andreas W. Gold, Eilin Rast, Anke Spura, Kayvan Bozorgmehr

**Affiliations:** 1 Sektion Health Equity Studies und Migration, Abteilung für Allgemeinmedizin und Versorgungsforschung, Universitätsklinikum Heidelberg, Im Neuenheimer Feld 130.3, 69120 Heidelberg, Deutschland; 2 Bundeszentrale für Gesundheitliche Aufklärung, Köln, Deutschland; 3 AG Bevölkerungsmedizin und Versorgungsforschung, Fakultät für Gesundheitswissenschaften, Universität Bielefeld, Bielefeld, Deutschland

**Keywords:** SARS-CoV-2, Migration, Infektionsschutz, Gesundheitliche Aufklärung, Öffentliche Gesundheit, SARS-CoV-2, Migration, Infection control, Health education, Public health

## Abstract

**Hintergrund:**

Die Eindämmung der COVID-19-Pandemie in Sammelunterkünften für Geflüchtete ist für die Wahrung deren körperlicher und psychischer Gesundheit enorm wichtig. Unklar ist, welche Maßnahmen in diesem Setting ergriffen werden, um das Infektionsrisiko zu senken, zusätzliche Stressoren in der Pandemie zu minimieren und über ergriffene Maßnahmen aufzuklären.

**Ziel:**

Situationsanalyse der Maßnahmen, die zur Prävention und Eindämmung des SARS-CoV-2-Virus in Sammelunterkünften für Geflüchtete ergriffen wurden, sowie Identifizierung von Unterstützungsbedarfen der Aufnahmebehörden.

**Methoden:**

Qualitative Interviewstudie mit 48 für die Unterbringung von Geflüchteten zuständigen Ansprechpartner*innen in den Aufnahmebehörden. Einzelinterviews wurden wörtlich transkribiert und mittels Framework-Analyse ausgewertet.

**Ergebnisse:**

In Bezug auf Maßnahmen des Infektionsschutzes, gesundheitlicher Information und Aufklärung, sozialer und gesundheitlicher Angebote, Testung auf SARS-CoV‑2 und Quarantäne zeichnet sich ein heterogenes Bild ab. Zur Abstimmung und Durchführung der Maßnahmen erwies sich eine effektive intersektorale Kooperation als besonders wichtig. Unterstützungsbedarfe bestehen in der Verbesserung der Unterbringung, dem vermehrten Einsatz von Sprachmittler*innen sowie der lokalen Stärkung gesundheitlicher Fachexpertise.

**Fazit:**

Aufgrund der hohen Anzahl an Akteur*innen und der Komplexität von Strukturen und Prozessen übernehmen Aufnahmebehörden ad hoc essenzielle Aufgaben des Infektionsschutzes, für die sie unzureichend aufgestellt sind. Für die Eindämmung der Pandemie sind eine settingspezifische Bündelung fachlicher Empfehlungen und Information auf Bundesebene sowie deren lokale Translation durch die proaktive Einbindung des öffentlichen Gesundheitsdienstes unabdingbar.

**Zusatzmaterial online:**

Zusätzliche Informationen sind in der Online-Version dieses Artikels (10.1007/s00103-021-03284-2) enthalten.

## Abstract (en)

**Background:**

The containment of the COVID-19 pandemic in collective accommodation centres is crucial to maintain the physical and mental health of refugees. It is unclear what measures have been taken by authorities in this setting to reduce the risk of infection, minimise stressors for refugees during the pandemic and communicate containment measures.

**Objectives:**

Assessment of measures that have been taken to prevent and contain SARS-CoV‑2 in collective accommodation for refugees and identification of support required by authorities.

**Methods:**

Qualitative interview study with 48 representatives responsible for the reception and accommodation of refugees. Individual interviews were transcribed verbatim and evaluated using framework analysis.

**Results:**

We found substantial heterogeneity of measures taken to prevent infection, inform refugees, maintain social and health services, test for SARS-CoV‑2 and quarantine positive cases. Effective intersectoral cooperation proved to be particularly important for coordination and implementation of measures. Need for support was expressed with regard to the improvement of infrastructure, opportunities to work with language interpreters and stronger involvement of local health experts.

**Conclusion:**

Amidst multiple actors and the complexity of structures and processes, the admission authorities have been taking on essential responsibilities related to infection control on an ad hoc basis, without being sufficiently positioned to do so. In order to further contain the pandemic, a strengthening of centralised, setting-specific recommendations and information as well as their translation through the pro-active involvement of the public health authorities at the local level are essential.

## Hintergrund

Seit Beginn der COVID-19-Pandemie kam es in Deutschland in Sammelunterkünften (SU) für Geflüchtete zu 199 Ausbrüchen (Stand: 11.08.2020) in Zusammenhang mit laborbestätigten SARS-CoV-2-Infektionen, die an das Robert Koch-Institut gemeldet wurden (RKI; [1]). Obwohl diese nur knapp 2,5 % aller 7864 an das RKI übermittelten SARS-CoV-2-Ausbrüche ausmachten, zählte das Infektionsumfeld der SU mit einer durchschnittlichen Fallzahl von ca. 21 Infizierten pro Ausbruch zu den größten, noch vor Alten- und Pflegeheimen [1]. In einzelnen Einrichtungen wurde trotz Eindämmungs- und Quarantänemaßnahmen bei mehr als 50 % der Bewohner*innen eine Infektion mit SARS-CoV‑2 nachgewiesen [2].

Besondere Fürsorge und Schutz für Geflüchtete ist ableitbar von internationalen und EU-weiten Rechtsnormen zur Wahrung eines angemessenen Lebensstandards, zu adäquater Unterbringung und Zugang zu gesundheitlicher Versorgung sowie der Wahrung des individuellen Rechts auf Vorbeugung und Bekämpfung epidemischer Erkrankungen [2]. Aufgrund ihrer Zuständigkeit für die Unterbringung und Sicherstellung der medizinischen Versorgung von Geflüchteten [3, 4] sind die oberen und unteren Aufnahmebehörden im Zuge der COVID-19-Pandemie auch in der Pflicht, Infektionsschutzmaßnahmen umzusetzen.

Diese Aufgabe wird durch den föderalen Prozess der Unterbringung Geflüchteter (gem. §§47, 53, 56 AsylG, §2 AsylbLG) und die Sammelunterbringung von Geflüchteten auf engstem Raum jedoch erschwert [5]. In Deutschland lebten Ende 2018 ca. 200.000 Geflüchtete in SU [6]. Dazu gehören sowohl Erstaufnahmeeinrichtungen (EA) der Länder als auch Gemeinschaftsunterkünfte (GU) der Kreise. Üblicherweise werden in SU mehrere Hundert Geflüchtete mit gemeinschaftlich genutzten Schlafräumen, Sanitäreinrichtungen und Kochmöglichkeiten untergebracht. Etwa die Hälfte aller Geflüchteten wohnte 2016 in Deutschland in SU mit durchschnittlich 11 qm Wohnfläche pro Person [7]. So sind angesichts der Unterbringungssituation die Empfehlungen zur Hygiene und zur physischen Distanzierung erschwert bis unmöglich umsetzbar. Die gängige Praxis der Verlegung Geflüchteter zwischen Unterkünften und der damit einhergehende behördliche Zuständigkeitswechsel stellen eine weitere Herausforderung für den Infektionsschutz dar.

Für Geflüchtete, welche durch traumatisierende Erfahrungen vor oder während der Flucht bereits überproportional häufig psychische Belastungen aufweisen, können Isolations- und Quarantänemaßnahmen zusätzliche Belastungen bedeuten [8, 9]. Die gesundheitliche Versorgung ist durch ein Zusammenspiel verschiedener Akteur*innen auf diversen föderalen Ebenen gekennzeichnet [4]. Somit stellt die COVID-19-Pandemie die Aufnahmebehörden sowie die Strukturen und Institutionen der Infektionskontrolle vor eine besonders komplexe Aufgabe, für die es aufgrund der fehlenden Berücksichtigung Geflüchteter im nationalen Pandemieplan [2] keine bundesweiten Vorgaben gab. Zwar hat das RKI Mitte Juli 2020 Empfehlungen für die Prävention und das Management von COVID-19-Erkrankungen in Aufnahmeeinrichtungen und Gemeinschaftsunterkünften herausgegeben [10], jedoch ist unklar, wie die lokalen Aufnahmebehörden in der Zeit davor, seit Nachweis des ersten SARS-CoV-2-Falls in Deutschland (28.02.2020), gearbeitet haben, um mit den diversen Herausforderungen des Ausbruchsgeschehens im Fluchtkontext umzugehen.

Ziel dieser qualitativen Studie ist eine Situationsanalyse der Regelungen und Maßnahmen auf lokaler Ebene, die zur Prävention und Eindämmung der SARS-CoV-2-Ausbreitung in SU für Geflüchtete ergriffen wurden. Es stellen sich 3 Fragen: Welche Maßnahmen wurden zum Infektionsschutz, zur Prävention von psychischen Belastungen und zur Aufklärung ergriffen? Auf welchen Informationen und Grundlagen basieren die Maßnahmen? Welche Bedarfe liegen seitens der Aufnahmebehörden vor?

Im Folgenden wird zunächst die methodische Vorgehensweise dieser Interviewstudie erläutert. Die Ergebnisse der geführten Interviews werden in 3 thematischen Abschnitten berichtet: Heterogenität der getroffenen Maßnahmen, intersektorale Zusammenarbeit und Verknüpfung zentraler Vorgaben mit lokaler Expertise. Abschließend werden die Ergebnisse mit Blick auf die Forschungsfragen diskutiert und Handlungsempfehlungen abgeleitet.

## Methoden

Es wurden semistrukturierte telefonische Leitfadeninterviews mit Akteur*innen aus EA und GU geführt (Zeitraum: 01.05.–31.07.2020). Der Leitfaden (siehe Onlinematerial) beinhaltete wesentliche Dimensionen der Prävention und Gesundheitsförderung, Infektionskontrolle und intersektoralen Zusammenarbeit. Maßnahmen der Sozialberatung, Alltagsbetreuung und Gesundheitsversorgung während der Pandemie waren aufgrund ihrer großen Bedeutung für die psychische Gesundheit Geflüchteter ein weiterer, essenzieller Bestandteil der Interviews. Nach Zustimmung der Innenministerien sowie Landkreistage wurden die für die Unterbringung Geflüchteter zuständigen Ansprechpartner*innen in den Sozial‑, Migrations- oder Ordnungsämtern (GU) bzw. in den Landesbehörden (EA) zum Interview eingeladen. Diese wurden über bestehende Forschungsnetzwerke zur Fluchtmigration bundeslandübergreifend rekrutiert.

Auf Ebene der EA konnten über ein überregionales Forschungsnetzwerk 18 Teilnehmer*innen aus 8 Bundesländern rekrutiert werden. Auf GU-Ebene fokussierte sich die Datenerhebung auf ein Flächenland mit einer hohen Flüchtlingszuweisung (nach Königsteiner Schlüssel, der die Aufteilung des Länderanteils bei gemeinsamen Finanzierungen regelt) und großen regionalen Strukturunterschieden. In diesem Bundesland wurden mit 27 Teilnehmer*innen Interviews geführt. Durch gezielte Rekrutierung von 3 Akteur*innen aus 3 weiteren Bundesländern wurden Interviews auf mögliche Sättigungseffekte überprüft. Die Interviews dauerten 25–105 min und wurden jeweils zu zweit geführt (von LB, RJ, CP, AG, ER).

Die Telefoninterviews wurden nach Information und schriftlicher Zustimmung digital aufgezeichnet, als Volltexte transkribiert, pseudonymisiert und mit MAXQDA Version 20 mittels der Framework-Analysemethode [11] in 2 Schritten qualitativ ausgewertet: Zunächst wurde in einem induktiv-deduktiven Verfahren auf Grundlage von Interviewnotizen ein Codesystem zu zentralen Themenbereichen entwickelt (siehe Onlinematerial) und dieses unter regelmäßigem Austausch auf alle Transkripte angewendet. Im zweiten Schritt wurden für jeden Themenbereich mittels einer Übersichtsmatrix (inkl. Information zu Art, Größe und Anzahl der SU; Bundesland; vorliegende Coronafälle) über die Interviews hinweg wichtige zusätzliche Informationen zu Mustern getroffener Maßnahmen und geäußerter Unterstützungsbedarfe gewonnen. Dabei wurden geäußerte Herausforderungen einem entsprechenden Handlungsbedarf gegenübergestellt und beispielhafte Zitate des jeweils zugeordneten Codes tabellarisch illustriert.

## Ergebnisse

Die 48 Studienteilnehmer*innen waren für insgesamt 441 SU und 37.009 Geflüchtete zuständig und hatten zwischen einer und 98 SU in ihrer Verantwortung. Während 23 Teilnehmer*innen zum Interviewzeitpunkt noch keine bestätigten COVID-19-Fälle hatten, berichteten die verbleibenden 25 über Fallzahlen zwischen einem bis über 400 infizierten Bewohner*innen. Die zweistufige Auswertung ergab 3 zentrale Themen, denen die folgende Ergebnisdarstellung in ihrer Struktur folgt: Heterogenität der Maßnahmen, intersektorale Zusammenarbeit, Verknüpfung zentraler Informationen mit lokaler Expertise.

### Heterogenität der getroffenen Maßnahmen

Es zeichnete sich eine hohe Heterogenität in der Aufklärung der Bewohner*innen, den allgemeinen Infektionsschutzmaßnahmen, dem Umgang mit sozialen und gesundheitlichen Angeboten sowie Test- und Quarantänemaßnahmen ab.

#### Gesundheitliche Information und Aufklärung

Die Teilnehmer*innen berichteten, dass Bewohner*innen aller Einrichtungen Informationen zum neuartigen Coronavirus und zu empfohlenen Verhaltensweisen erhielten. Dabei wurden unterschiedliche Informationswege genutzt. Allgemeine Informationen zum Virus und Hygienemaßnahmen wurden tendenziell schriftlich und, wenn vorhanden, über multimediale Informationswege (vor allem soziale Medien, aber auch vereinzelt eigens entwickelte Apps und Videos) kommuniziert. Mündliche Informationswege dienten vor allem zur Sensibilisierung sowie der Informierung zu einrichtungsspezifischen und personenbezogenen Infektionsschutzmaßnahmen (z. B. Quarantäne). Vereinzelt boten ehrenamtliche Multiplikator*innen Schulungen an oder informierten die Bewohner*innen mündlich. Einige Teilnehmer*innen betonten die hohe Relevanz mündlicher Information, um Fehlinformation, Panik oder Verharmlosung entgegenzuwirken: „Bewohner sind mal […] vorstellig geworden, dass sie gesagt haben ‚Wir haben in Afrika Ebola überlebt, was soll uns hier irgend so ein deutscher Virus‘ und denen wurde dann eben gesagt ‚Ja, auch *euch* betriffts, auch *euch* kann es schädigen‘ also es geht […] um Sensibilisierung, vor allem, um übertriebenen Reaktionen entgegenzuwirken, in dem man einfach aufklärt“ (12LK).

Als Informations- und Materialquellen wurden Rechtsverordnungen, das Informationsangebot des RKI, die Bundeszentrale für gesundheitliche Aufklärung (BZgA), die Integrationsbeauftragte der Bundesregierung sowie ergänzende landes- und kreisspezifische Informationsangebote aufgeführt.

Es zeigte sich, dass der Einsatz von Übersetzungen und Sprachmittler*innen sehr heterogen erfolgte. Meistens wurde auf bestehende Übersetzungen zurückgegriffen, seltener wurden zusätzliche Übersetzungen angefertigt (z. B. einrichtungsspezifische Informationen, behördliche Anordnungen). In Einzelfällen wurden professionelle Sprachmittler*innen eingesetzt. In der Regel kam dabei überwiegend bestehendes Personal (v. a. aus der Sozialbetreuung und dem Sicherheitsdienst) zum Einsatz.

Die Informierung der Bewohner*innen ist den Teilnehmer*innen zufolge vor allem aufgrund der Informationsfülle und -komplexität herausfordernd. Daher äußerten sie den Bedarf nach einer zentralen Auswahl von Informationsangeboten, die auf den Kontext der Unterkünfte angepasst sind (z. B. in leichter Sprache und/oder mit Piktogrammen), sowie einer stärkeren Einbindung von Sprachmittler*innen für die persönliche Kommunikation (Tab. 1).

**Table 1:** 

	Herausforderung	Handlungsbedarf
Aufklärung Bewohner*innen: Fülle an Information	**Fülle an möglichen Informationsquellen und Herausforderung, geeignete Materialien auszuwählen** „Es gibt dann auf einmal […] ein reichhaltiges Angebot, das wächst ja jeden Tag an Informationen. Und auch bei uns, die [Organisationen] untereinander, man tauscht sich aus. Jeder erfindet dann das Rad neu und man hat dann eine regelrechte *Informationsflut*. Und das führt dann meistens dazu, dass so eine Einrichtung schnell aussieht wie so eine Litfaßsäule, weil man 50 verschiedene Aushänge hat, die aber alle letztendlich das Gleiche *meinen*“ (35EA)	**Bedarf an zentraler Bündelung settingspezifischer und wissenschaftlich begutachteter Informationsangebote für Geflüchtete** „Also das RKI stellt dann Informationen bereit, aber halt auch nur die Informationen des RKI. Da wäre eine größere und stärkere Koordination und eine stärkere Recherche, die dann vielleicht nicht jedes Bundesland einzeln machen müsste oder nicht jede Gemeinde einzeln machen müsste oder noch stärker jede einzelne Ambulanz selber machen müsste, sondern dass man darauf zugreifen könnte und da sich relativ sicher sein könnte, dass da die validen Patienten und auch sonstigen Informationen sind“ (28EA)
Aufklärung Bewohner*innen: Verständlichkeit	Informationsquellen verwenden schwierige Sprache, schriftliche Informationen deshalb schwer verständlich für Bewohner*innen mit niedrigem Bildungsgrad „[…] die meisten Informationsschreiben, die es da so gab auch von Landesseite, von den Gesundheitsämtern, die waren halt für Sie und mich gedacht, wir, die der deutschen Sprache komplett mächtig sind, und da haben wir halt bei manchen gemerkt: okay, die sind so einfach nicht verwendbar“ (12LK) „Also *geschrieben* hat man sehr viel bekommen, aber wir haben einfach gemerkt, dass das Geschriebene flächendeckend nicht überall wahrgenommen wird. Also auch mit Aushängen oder sonstiges, das funktioniert eben nicht ganz so gut“ (38SK)	**Bereitstellen von Informationsmaterial in leichter und bildlicher Sprache** „[…] wir machen das ohnehin so […], dass wir einfache Sprache benutzen und […] als Grundlage für die Übersetzung überhaupt nehmen und, wo es geht, auch mit Piktogrammen arbeiten“ (08SK)
		Stärkung der mündlichen Informierungsmöglichkeiten mit Sprachmittler*innen „[…] klar haben wir auch relativ hohe Analphabetenquote, dafür gibt es dann aber die Sozialberatung oder Leute mit Sprachmittlern, […] sodass man den Geflüchteten halt auch wirklich die Allgemeinverfügung erklären kann in ihrer Sprache, sodass sie es *verstehen*, das war uns auch ganz wichtig, dafür ansprechbar zu sein“ (44EA)
Infektionsschutz: fehlende räumliche Kapazitäten	**Bedarf an zusätzlichen räumlichen Kapazitäten zur Entzerrung der Belegung** „Soziale Distanzierung, das ist brutal schwer, weil […] [es] sind pro Gebäude hundert Leute untergebracht. Das heißt, je Stockwerk 30 bis 40 Leute. […] Die benutzen die gleiche Duschanlage, und die benutzen die gleichen Waschräume. 30, 40, manchmal 50 Leute. Und den Leuten dann zu sagen, ‚Ihr dürft euch auf dem Gelände nur zu zweit oder im Familienverband aufhalten‘, das ist sehr, sehr schwer zu vermitteln“ (31EA)	**Bedarf eines langfristigen räumlichen Kapazitätsaufbaus mit stringenten Qualitätsstandards** „Die grundsätzlichen Rahmenbedingungen müssen verbessert werden, weil es gibt eine Vorgabe bei der Asylunterbringung von 7 Quadratmetern für jede Person und eine Auslastung von 80 % der Unterkünfte, […] und das zeigt sich in solchen Pandemien, dass […] der Schuss nach hinten losgeht in dem Moment, weil man nämlich keine Kapazitäten hat, darauf zu reagieren“ (4SK) „Ich hätte in meinen Gemeinschaftsunterkünften auf jeden Fall gerne mehr sanitäre Anlagen. Und es hat sich bei Corona halt gezeigt, dass es dringend erforderlich ist, das war es aber auch vorher schon. Die meisten eingerichteten Gemeinschaftsunterkünfte sind ja schon jahrelang in Betrieb oder in der Flüchtlingshochzeit relativ kurzfristig eingerichtet worden und bieten deshalb meiner Meinung nach nicht immer einen erforderlichen Sanitärstandard“ (46SK)
Infektionsschutz: Verlegung	**Für Kreise: Problematik durch Zuverlegung aus EAs, aber Weiterverlegung in Gemeinden nur bedingt möglich** „Umgekehrt hat die Politik natürlich auch gesagt ‚Zweifellos finden die Zuweisungen weiterhin statt‘ also eher mehr als weniger […] Das bedeutet für uns klar wenn wir sagen ‚Wir wollen ein bisschen vorsichtig sein‘, dann brauchen wir aber umso schneller wieder eine zweite und eine dritte Unterkunft“ (06LK)	
Gesundheitliche Versorgung: Koordination	**Herausforderung, mit geänderten Abläufen, aber auch Ängsten externer Gesundheitsdienstleister umzugehen** „Ja natürlich, also es war schwieriger für unsere Bewohner, zum Arzt zu kommen, weil die Ärzte natürlich auch ihre Abläufe geändert hatten, weil es schwieriger war, einen Termin zu bekommen […] dann auch noch die Sprachbarriere dazu, und was bei uns ein großes Problem war, da weiß ich bis heute noch nicht wo es her kam – die Landesaufnahmestelle wurde als Hotspot des Coronavirus betitelt […] – das war nicht der Fall und da hatten viele Ärzte einfach Angst, sich zurückgezogen und haben die Behandlung teilweise wirklich verweigert“ (32EA)	**Ausweitung der Aufgaben des Einrichtungspersonals (z.** **B. Sozialarbeiter) um gesundheitliche Vermittlung und Koordination bzw. Einrichtung spezifischer koordinierender Stelle** „Krankheitsfälle werden von den Sozialarbeitern auch engmaschig betreut, ähm mir fällt jetzt gerade ein Fall ein, in der Anschlussunterbringung hatten wir bestätigte Verdachtsfälle, da war eine Frau dabei, die krebskrank ist und die auch an ihre Medikamente hätte kommen müssen, die zusätzlich dann auch unter Quarantäne gesetzt war, da hat sich dann der Sozialarbeiter darum gekümmert, der war dann in der Uniklinik in [Stadt48] hat die Medikamente organisiert, ansonsten haben wir das immer auch abgefragt also die Termine konnten eigentlich auch wahrgenommen werden zu den Ärzten“ (17LK) „Also bisher war es immer so auch, dass die Sozialbetreuung immer eng involviert war, wenn der Flüchtling Symptome gezeigt hat, und in der Regel hat das die Sozialbetreuung auch mit dem Hausarzt geklärt. Und der Hausarzt ruft dann in der Regel auch wieder die Sozialbetreuung zurück und gibt denen die Information, äh das Testergebnis wird da mitgeteilt“ (05LK*)*
Testung: Informationsweitergabe	Inkenntnissetzung der Einrichtungsleitung über positive Testergebnisse bei Bewohner*innen „Wenn natürlich jemand zum Gesundheitsamt geht, und sich dort testen lässt ohne das zu kommunizieren, wird es zweifellos schwieriger sein daran zu kommen, aber häufig ist es so, dass das dann auch das Umfeld dort irgendwie mitbekommt wenn es jemandem nicht gut geht oder die Menschen wenden sich direkt an unsere Mitarbeiter vor Ort und sagen ‚Husten, Halsweh – ich will mich testen lassen‘ – dann bekommen wir entsprechend Rückmeldung auch vom Gesundheitsamt“ (02SK)	
Quarantäne: Schutzausrüstung	**Knappheit an Schutzausrüstung** „Problem war am Anfang natürlich auch, Schutzmaterial […] Masken, Schutzanzüge, auch für die Hausverwalter zu bekommen, da sind uns auch Lieferungen zugesagt worden, die man dann wieder umgeleitet hat an die Krankenhäuser, was irgendwo auch verständlich ist“ (43LK)	**Effektive behördliche Unterstützung und Führung; Einbindung von Geflüchteten in Gesamtstrategien der lokalen Behörden** „Wir haben natürlich auch einen Landrat, der das volle Vertrauen in uns hat, und wenn wir was brauchen, […] er geht voran in der Corona-Krise, also muss ich wirklich sagen, haben wir ein sehr gutes Oberhaupt“ (18LK)
Zusammenarbeit Gesundheitsamt: Ressourcenknappheit	**Unzureichende Erreichbarkeit und Einbindung des Gesundheitsamtes in Strategien vor Ort** „Ich sage es mal so: Wenn du eine Einrichtung hast, die unter Gesamtquarantäne ist, die dann auch in der [Presse] als Corona-Hotspot bezeichnet wird, […] [aber] das Gesundheitsamt war nie da bei uns und hat mit uns geredet über die Themen […], dann denke ich, es spricht für mich schon ein bisschen Bände, was die Zusammenarbeit angeht“* (anonym)* „Die Einzigen, die mein vollstes Mitleid verdienen, sind die Gesundheitsämter, weil die teilweise wirklich *dermaßen* abgesoffen sind. So war unser Eindruck, dass der einzige *Wunsch* vielleicht gewesen wäre, […] weiterhin diesen engen *Draht* zu denen zu haben. Das war aber rein menschlich nicht möglich“ (35EA)	**Stärkung des öffentlichen Gesundheitsdienstes und engere Einbindung dessen gesundheitlicher Expertise** „Also was ich mir grundsätzlich wünschen würde ist natürlich ein personell noch besser ausgestattetes Gesundheitsamt […] dass die ein bisschen mehr Ruhe hätten, um das zu machen, das wäre natürlich sehr schön, weil dann könnte man da auch mit einem ruhigeren Gewissen anrufen und das nochmal in Ruhe durchsprechen und so weiter, die nehmen sich schon die Zeit, alles gut, aber ich weiß auch, unter was für einem Druck die stehen“ (09LK) „Da nochmal eine individuelle Info wäre schön gewesen weil es kam wie gesagt diese Info […] wo auch drin steht was die in den Landeserstaufnahmeeinrichtungen machen, ja das stimmt schon, aber, das ist so, ‚Hier hast du acht Seiten, lies das durch und guck was für dich wichtig ist‘ so eine, ein Ticken individuellere Info wäre schön gewesen“ (06LK*)*
Empfehlungen: lokale Diversität	**Unterschiedliche infrastrukturelle Bedingungen** „Jeder Landkreis ist aufgrund seiner Infrastruktur anders, muss da selber überlegen, wie kann er vorbeugend tätig werden“ (25LK)	
Quarantäne: Planbarkeit	**Planbarkeit der Versorgung während der Quarantäne schwierig** „Ein Problem wird immer sein, wenn Personen unter Quarantäne gestellt werden, die dann zu versorgen. Das kann man organisieren mit einem Lieferservice, mit einem Einkaufsdienst, häufig ist das Problem dann allerdings auch […] die finanzielle Seite, wie kommen sie dann an ihr Geld – […] das gestaltet immer das System ein wenig schwierig hier Menschen zu versorgen“ (4SK) „Bis heute haben wir da einfach keine verbindliche Aussage, weil das Gesundheitsamt auch sagt, das müssen sie im Einzelfall entscheiden und das müssen wir dann natürlich auch so hinnehmen“ (03SK)	**Möglichkeit der Vorabplanung mit dem Gesundheitsamt** „[…] in einigen Landkreisen hätte ich mir die Zusammenarbeit mit den Gesundheitsämtern noch enger und transparenter vorgestellt, […] die klassische Frage: ‚Gibt es einen Plan für unsere Unterkunft, wenn wir einen Fall haben?‘ die wurde dann oft damit beantwortet mit ‚naja dann machen wir einen Plan‘ – da hätte ich mir gewünscht, das man sich da auch einfach mal mit dem Gesundheitsamt vielleicht auch mal richtig zusammengesetzt hätte und einen Plan zusammen entwickelt hätte“ (45LK)
Quarantäne: Informationsbedarf	**Fehlende Information und Expertise für die Personen, welche die Quarantäne umsetzen** „Was so Begriffe wie, Isolierung, Kohortenisolierung das sind alles Sachen, da wird man mit konfrontiert, dann denkt man ‚Kohortenisolierung: Was ist das? Wie mache ich das? Was muss ich beachten?‘, also in dieser Richtung, einfach […] Unterstützung, für den Verwaltungsmensch vor Ort“ (32EA) „Ich bin kein Mediziner, ich maße mir da kein Urteil an, uns hat das ein bisschen verwundert, weil wir dachten, es wäre sinnhafter, wenn drei Verdachtsfälle, in drei verschiedenen Unterkünften auftauchen, dass man die lieber dann zusammen rausnimmt, in eine Unterkunft nimmt“ (03SK)	**Zentrale Empfehlungen für das Vorgehen im Setting der Flüchtlingsunterkünfte, eingebettet in eine Gesamtstrategie auf Bundesebene** „Was ich mir gewünscht hätte, im Vorfeld, wären Pläne gewesen, der Bundesbehörden, wie man eigentlich in solchen Einrichtungen vorgehen muss. Dass ich mir nicht tausend Stunden Gedanken machen muss, und *wirklich* tausend Stunden, um das mir zu erarbeiten, sondern ich hätte mir aus dem Bundes*gesundheits*ministerium irgendwie einfach einen Katalog gewünscht, wo drinsteht, was mache ich bei der Pandemie [in einer Aufnahmeeinrichtung], und dann arbeite ich einfach das ab“ (39EA) „Du musst dir eigentlich alles selber aus dem Finger saugen und du hast wenig Vorgaben gehabt von Ministerien, von Gesundheitsverwaltung ähm. […] Das hätte uns schon geholfen, wenn wir Vorgaben gehabt hätten zu *Beginn*. Also wir haben ja uns frühzeitig drum gekümmert […]. Das hätte sehr geholfen, wenn wir da, Vorgaben gehabt hätten, wie verhalten wir uns, wie gehen wir mit Positiven, wie gehen wir mit Negativen um“ (31EA)
Zusammenarbeit Gesundheitsamt: uneinheitliche Vorgehensweisen	**Unterschiedliche Gesundheitsämter haben uneinheitliche Vorgaben und Vorgehensweisen** „Da gab es einfach sehr unterschiedliche Vorgehensweisen. Manche wollten zum Beispiel die Kontaktpersonenermittlung *un*bedingt selbst machen, andere haben gesagt: ‚Das kommt überhaupt nicht in Frage […]‘. Dann wurde auch die Anordnung von Reihentestungen unterschiedlich gehandhabt. In manchen Unterkünften hatten wir’s, in anderen nicht, dann was auch unterschiedlich war, war eine abschließende Testung, nach Ablauf der Quarantäne, manche haben’s angeordnet, andere NICHT, das hat uns vor Herausforderungen gestellt“ (42EA)	
Empfehlungen: Umsetzbarkeit	**Allgemeine Empfehlungen lassen sich im Flüchtlingskontext nicht umsetzen** „[…] dann hat sich gezeigt, dass das in den Unterkünften natürlich nicht möglich ist […] wir packen irgendwo unsere Hausmeister zusammen und wir packen irgendwo die Sozialarbeiterinnen zusammen und die gehen dann raus, wenn irgendwo, was weiß ich, gemeldet wird ‚da läuft jetzt Wasser aus der Steckdose‘ oder ‚da gibt es jetzt einen Tumult der Bewohner‘, das funktioniert nicht“ (02SK)	

^a^Die Tabelle enthält beispielhafte Zitate, die dem jeweiligen Code zugeordnet wurden

#### Infektionsschutzmaßnahmen in SU

Laut der Teilnehmer*innen wurden als allgemeine Infektionsschutzmaßnahmen Mund-Nasen-Bedeckungen (MNS) für Mitarbeitende und Bewohner*innen ausgegeben sowie teils Desinfektionsmittel und Seifenspender bereitgestellt. Vereinzelt wurden Änderungen der Reinigungsintervalle und vermehrte Flächendesinfektion berichtet. Das Tragen eines MNS für Bewohner*innen sei in den meisten Unterkünften nicht verpflichtend bzw. nur räumlich begrenzt gewesen. Während in einigen Einrichtungen MNS bereits im Februar/März beschafft oder über Nähprojekte selbst produziert wurden, erfolgte eine Ausgabe anderswo erst nach behördlicher Maskenpflicht.

Abstandsregelungen galten für Gemeinschaftsanlagen wie Küchen, Sanitäranlagen, Spielplätze und Wartebereiche. SU mit Kantinen stellten vielerorts auf „Folienessen“ (27EA) zum Verzehr auf den Zimmern um. Zur weiteren Kontaktreduzierung wurden, insbesondere in größeren SU, Besuchsverbote ausgesprochen – „dass eben nur tatsächlich Bewohner oder eben Mitarbeiter vor Ort in die Unterkünfte dürfen, um dieses Infektionsrisiko zu reduzieren“ (38SK).

Etwa die Hälfte der Interviewten veranlasste eine Entzerrung der Belegung in den SU. In 2 Bundesländern erfolgte dies primär durch fortgesetzte Weiterverlegungen von EA in GU. In anderen Bundesländern und auf Kreisebene wurden Geflüchtete innerhalb bestehender Strukturen umverteilt. Auf GU-Ebene wurde nur vereinzelt die Schaffung zusätzlicher Kapazitäten berichtet, auf EA-Ebene war dies öfters der Fall. Teilweise seien Personen mit einer erhöhten Vulnerabilität kontaktarm untergebracht oder prioritär aus den EA in GU oder spezielle Schutzunterkünfte verlegt worden.

Während physische Distanzierung in einigen, kleineren Unterkünften mit „Wohnungscharakter“ (9LK) gut möglich war, war sie in großen, dicht belegten SU mit gemeinschaftlichen Koch- und Sanitäreinheiten besonders herausfordernd. Als Unterstützungsbedarf wurde vorwiegend eine allgemeine Verbesserung der Kapazitäten und der baulichen Bedingungen in den SU genannt (Tab. 1).

#### Anpassungen sozialer Unterstützungsangebote

Soziale Unterstützungsangebote wurden je nach Angebotsart unterschiedlich an Distanzierungs- und Infektionsschutzmaßnahmen angepasst. Gruppenangebote und Kinderbetreuung wurden (teils temporär begrenzt) eingestellt sowie Spiel- und Sportplätze geschlossen. Teilweise wurden professionelle und ehrenamtliche Beratungs‑, Freizeit- und Bildungsangebote komplett ausgesetzt. Daraus resultierte ein Wegfall der Tagesstruktur für die Bewohner*innen, was auch in Bezug auf die psychische Belastung als Stressor betrachtet wurde: „Es ist brutal schwierig, wenn die komplette Tagesstruktur wegbricht in Form von Kinderbetreuung, von Sprachkursen, von Integrationskursen, von Arbeitsgelegenheiten für die Gemeinnützigen, Sportgelegenheiten ebenso. Und es ist quasi nichts da, was das auf irgendeine Art und Weise kompensieren kann“ (31EA). Vereinzelt wurde als Ausgleich auf Individualangebote, auf Bildungsangebote mit schriftlichen Lernmaterialien oder auf Onlinelernformate umgestellt.

Auch die Alltagsbetreuung, das Integrationsmanagement sowie die Sozial- und Verfahrensberatung stellten sich unterschiedlich auf die veränderten Umstände ein. In einigen Einrichtungen blieben Sozialarbeiter*innen weiterhin vor Ort, wobei Beratungsangebote unter Berücksichtigung von Infektionsschutzmaßnahmen (1,5–4 m Abstand, MNS-Pflicht, Schutzausrüstung, Plexiglasscheiben) oder eingeschränkt bei Neuzugängen und in Notfällen stattfanden. Teils wurden offene Sprechzeiten reduziert oder eingestellt bzw. durch Terminvergaben ersetzt, in externen Büros oder im Freien abgehalten.

Zwar wurden für Sozialberatung, administrative Aufgaben und behördliche Angelegenheiten vermehrt postalische, elektronische und telefonische Kommunikationswege (einschließlich Messengerdienste) genutzt, jedoch wiesen einige Teilnehmer*innen darauf hin, dass diese Ausweichmöglichkeiten Inanspruchnahmebarrieren erzeugen können. Persönliche Kontakte seien gerade während der Coronapandemie wichtig, um auf Ängste, Beratungs- und Informationsbedarfe der Bewohner*innen einzugehen und somit die mögliche psychische Belastung in der Situation abzufedern: „Gerade in dieser Coronazeit, wo sich ja alle anderen zurückgezogen haben, sämtliche Migrationsberatungsstellen […] haben ihren Betrieb eingestellt, die Sozialämter haben ihre Häuser geschlossen und waren für die Bewohner auch nur noch telefonisch oder online zu erreichen was dann ja auch oft eine Barriere darstellt, auch eine sprachliche Barriere, also so dass wir zu Zeiten des ganzen Lockdowns auch die einzigen Ansprechpartner waren und da musste die Sozialberatung einfach weitergehen, […] das konnten wir nicht auch noch zurückfahren“ (45LK).

#### Anpassung gesundheitlicher Versorgungsangebote

Ein Großteil der Teilnehmer*innen gab an, weiterhin eine adäquate medizinische Versorgung gewährleisten zu können. Stellenweise wurden pandemiebedingte Herausforderungen berichtet, z. B. aufgrund wahrgenommener Mehrbedarfe der Bewohner*innen im Bereich psychosozialer Belastung, aufgrund von geänderten Abläufen oder aufgrund von Ängsten in umliegenden Praxen vor Ansteckungen durch die SU-Bewohner*innen. Daher wurden medizinische Angebote vor Ort teils spezifisch ausgeweitet sowie die koordinative und kommunikative Rolle der Soziarbeiter*innen in der medizinischen Versorgung gestärkt (Tab. 1). In einer Einrichtung wurde ein neues Programm zur psychosozialen Unterstützung initiiert. In einzelnen Fällen jedoch waren die Möglichkeiten, durch Anpassungen der Versorgung auf COVID-19 zu reagieren, aufgrund vorbestehender Problemlagen, wie zum Beispiel Ärztemangel, stark eingeschränkt.

#### Testung auf SARS-CoV-2

In Bezug auf die Testung von Personen mit klinischem Verdacht auf eine SARS-CoV-2-Infektion wurden unterschiedliche Vorgehensweisen sowie Herausforderungen berichtet. Bereits die Erfassung von Verdachtsfällen variierte. Es wurde sowohl von strukturierten Verdachtserfassungen berichtet (über die Einrichtungsambulanzen, mobile Teams zur Fiebermessung, Durchführung strukturierter Symptomabfragen, Fiebermessungen durch nichtärztliches Personal) als auch von Fallerfassungen in der Einrichtungsambulanz, durch nichtärztliches Einrichtungspersonal oder auf Initiative der betroffenen Bewohner*innen direkt durch die behandelnden Hausärzt*innen ohne Einbindung von Einrichtungspersonal.

Weitgehende Einheitlichkeit bestand lediglich im Bereich der Testung und 14-tägigen Isolation von neu registrierten Asylsuchenden. 11 Teilnehmer*innen der EA berichteten zudem, Testungen von Asylsuchenden vor Verlegung in die Anschlussunterbringung routinemäßig durchzuführen, in anderen war dies nur teilweise der Fall oder wurde prinzipiell abgelehnt. Darüber hinaus fanden in mehreren SU Reihentestungen statt. Diese erfolgten entweder nach Auftreten bestätigter SARS-CoV-2-Infektionen oder seltener präventiv aufgrund von hohen Infektionszahlen in anderen Aufnahmeeinrichtungen oder Pflegeheimen.

Als gemeinsame Herausforderung der verschiedenen Testungsanlässe und -abläufe zeichnete sich vor allem die Inkenntnissetzung der Einrichtungsleitung, d. h. die zeitgerechte Informationsweitergabe über positive Testergebnisse bei Bewohner*innen, ab. Vor allem in GU, in denen umliegende Praxen die Testungen ohne Beteiligung der Einrichtungsleitung oder Sozialarbeiter*innen durchführten, erfolgte die Informationsweitergabe nach Aussage der Interviewten teilweise nur unvollständig oder gar nicht (Tab. 1). Bei engen Kontakten zum Gesundheitsamt konnten positive Testergebnisse oft zeitnah weitergegeben werden, wobei in Einzelfällen die Weitergabe unter Verweis auf den Datenschutz (z. B. bei Personal der SU) nur verzögert stattfand.

#### Quarantäne- und Isolationsmaßnahmen

Bei Auftreten einer Infektion in der Unterkunft wurde von Quarantänemaßnahmen mit getrennter Unterbringung von Infizierten und Kontaktpersonen berichtet, entweder innerhalb der SU oder in einer speziell dafür vorgesehenen Unterkunft. Einige Teilnehmer*innen beschrieben, dass eine Trennung Infizierter untereinander nicht notwendig sei, da „es nicht darauf ankommt, ob er sich das zweite dritte vierte Mal infiziert“ (13EA).

Teilnehmer*innen, in deren SU noch keine Infektionen aufgetreten waren, nahmen oftmals an, dass es bei Auftreten eines Falles zu einer Gesamtquarantäne der Unterkünfte kommen würde, da dies in anderen SU der Fall war: „im Allgemeinen oder zu 99 %, so wie wir es mitbekommen haben, wird ja die gesamte Einrichtung unter Quarantäne gestellt“ (20LK). Jedoch wurde nur vereinzelt bzw. bei größeren Ausbruchsgeschehen von einer tatsächlich eingetretenen Gesamtquarantäne der Unterkünfte berichtet. Mehrere Teilnehmer*innen bewerteten die Gesamtquarantäne aus praktischen und ethischen Gesichtspunkten kritisch: „ganz klar ist, man sollte die Leute nicht vor-verurteilen, davon ausgehen, dass sich keiner daranhalten wird, und deswegen eine Unterkunft die unter Quarantäne steht einfach mal per se abschließen, ich finde daran kann Keiner Interesse haben“ (12LK).

Die Quarantänemaßnahmen wurden laut Teilnehmer*innen vielerorts durch Aufstellen von Bauzäunen und vermehrten Einsatz von Sicherheitspersonal durchgesetzt. Einige Teilnehmer*innen thematisieren hierbei die Unterschiede im Vergleich zur deutschen Regelbevölkerung: „diejenigen, die bei uns in Quarantäne sind oder in Isolation sind VIEL überwachter als Menschen, die sich in der Allgemeinbevölkerung anstecken“ (39EA). Manche Kreise entschieden sich deshalb gegen eine vermehrte Überwachung: „Die hatten keinen Sicherheitsdienst, weil wir auch gesagt haben: ‚eigene Verantwortung, so wie bei jedem Deutschen auch‘“ (23LK).

In den Interviews wurde ein sehr hoher Bedarf an Informationen und Expertise bei der frühzeitigen Planung sinnvoller, angemessener und praktikabler Quarantänemaßnahmen geäußert, um bei Auftreten einer Infektion oder im Ausbruchsfall in der SU adäquat reagieren zu können. Dies sei gerade deswegen von hoher Relevanz, da eine Ausgangssperre bzw. Quarantäne der gesamten Einrichtung zusätzliche psychische Belastungen unter Geflüchteten verursachen kann. Eine weitere Herausforderung entstand aus dem organisatorischen Aufwand, die Verpflegung mit Nahrungsmitteln, Betreuung, gesundheitliche Versorgung, aber auch z. B. die Müllabfuhr für die Bewohner*innen in der Quarantäne sicherzustellen. Hier wurde insbesondere auch die Beschaffung von Schutzausrüstung für Mitarbeitende im Quarantänebereich genannt, welche sich teils sehr schwierig darstellte und durch Organisationen des Katastrophenschutzes oder der Bundeswehr unterstützt werden musste. Um diesen Aufwand zu bewältigen, wurde eine frühzeitige Planung der konkreten Quarantänemaßnahmen gemeinsam mit dem Gesundheitsamt sowie die Klärung und ggf. Zentralisierung der Beschaffung als notwendig erachtet (Tab. 1).

### Intersektorale Zusammenarbeit

Trotz der zentralen Rolle der Aufnahmebehörden bei der Unterbringung Geflüchteter zeigte sich die hohe Relevanz einer effektiven intersektoralen Kooperation und Kommunikation zwischen den zahlreichen an der Flüchtlingshilfe beteiligten Akteur*innen. Zentral sind die Gesundheitsämter, aber auch örtliche Ordnungsämter und Polizeibehörden (z. B. bei der Umsetzung der Distanzierungs- und Quarantänemaßnahmen), zuständige Sozialbehörden sowie externe Dienstleister (z. B. mit der Sozialberatung beauftragt).

#### Zusammenarbeit mit Gesundheitsämtern

In der Zusammenarbeit mit den Gesundheitsämtern berichteten die Teilnehmer*innen von sehr unterschiedlichen Formen und Erfahrungen. Obwohl es mehrere Schnittstellen zur Zusammenarbeit zwischen Gesundheits- und Aufnahmebehörden gibt (Tab. 2), variierte die konkrete Kooperation lokal erheblich. Überwiegend wurde von praktischer Zusammenarbeit (z. B. im Rahmen der Ausbruchskontrolle) und seltener von konzeptioneller (z. B. präventiver) Zusammenarbeit mit den zuständigen Gesundheitsämtern berichtet.

**Table 2:** 

Konzeptionelle Zusammenarbeit	Praktische Zusammenarbeit
– Beratung der Aufnahmebehörden durch Gesundheitsämter – Erstellung von Hygienekonzepten – Erstellung eines prospektiven Quarantänekonzepts – Beratung zu Lockerungsmaßnahmen	– Testungen auf SARS-CoV-2 unter Bewohner*innen und ggf. Personal (Anordnung, Organisation, Durchführung) – Kontaktpersonenermittlung bei Verdachts- und Positivfällen – Erlass von Quarantäneanordnungen/-verfügungen auf Grundlage des Infektionsschutzgesetzes (IfSG; ggf. gemeinsam mit zuständiger Ordnungsbehörde) und Kommunikation an Betroffene – Präventions‑, Aufklärungsangebote der Gesundheitsämter für Bewohner*innen

Um den Herausforderungen im Rahmen der COVID-19-Pandemie gemeinsam begegnen zu können, wurde eine gute Erreichbarkeit des Gesundheitsamtes und eine gemeinsame Be- und Erarbeitung von spezifischen Maßnahmen als notwendig erachtet. Bereits etablierte und tragfähige Kooperationsstrukturen waren hierbei förderlich. Insbesondere auf Kreisebene erleichterten kurze Dienstwege innerhalb der Behörde, wie beispielsweise die Zugehörigkeit von Aufnahme- und Gesundheitsbehörde innerhalb eines Dezernats, die intersektorale Zusammenarbeit.

Ein überwiegender Teil der Teilnehmer*innen berichtete jedoch auch über vorübergehende oder dauerhafte Herausforderungen, die sich vor allem in Form unzureichender Erreichbarkeit sowie gänzlich fehlender Möglichkeiten des Austauschs und der Zusammenarbeit mit den jeweils zuständigen Gesundheitsämtern darstellten. Gleichwohl wurde durchgängig hohes Verständnis für die Arbeitsbelastung in den Gesundheitsbehörden geäußert. Entsprechend wurden vielfach Wünsche nach einer Stärkung des öffentlichen Gesundheitsdienstes (ÖGD) geäußert, um hierdurch die Zusammenarbeit zwischen den Aufnahme- und Gesundheitsbehörden intensivieren zu können. Einige der Teilnehmer*innen aus EA, die mit mehreren Gesundheitsämtern zusammenarbeiteten, berichten zudem von unterschiedlichen Herangehensweisen und Empfehlungen der jeweiligen Gesundheitsämter (z. B. bei Testverfahren, Kontaktpersonenermittlung). Daraus resultierten Unsicherheiten bei der Maßnahmenplanung und -umsetzung (Tab. 1).

#### Lokalpolitische Zusammenarbeit

Einige Teilnehmer*innen thematisierten explizit die Notwendigkeit einer engen und vertrauensvollen Kommunikation mit lokalen Akteur*innen, um in der erlebten Krisensituation eine effektive Zusammenarbeit zu gewährleisten. Hier wurde oft darauf verwiesen, dass diese Vertrauensbasis über mehrere Jahre der Zusammenarbeit und gerade auch durch die gemeinsame Arbeit im Jahr 2015 geschaffen wurde: „Also das Netzwerk hat funktioniert und das ist ein komplexes Netzwerk gewesen. Natürlich hat es nur deswegen funktioniert, weil wir das Vertrauensverhältnis schon vorher hatten aus der anderen Zeit“ (40EA).

Nahezu alle Teilnehmer*innen berichteten, dass die intersektorale Zusammenarbeit unter Pandemiebedingungen über Verwaltungs‑, Führungs- bzw. Krisenstäbe in ihrem behördlichen Umfeld gesteuert wurde. In diesen Strukturen seien die Teilnehmer*innen jedoch nur teilweise selbst eingebunden. Einige Teilnehmer*innen berichteten explizit, dass aufgrund der konkurrierenden Prioritäten im Zuge der Pandemie das Thema der Flüchtlingsunterbringung Fürsprache erforderte: „[…] wir mussten eben auch erstmal hinkriegen, dass man so registriert auf allen Ebenen, dass unsere Belange durchaus auch sehr wichtig sein könnten“ (12LK). Bei entsprechender Priorisierung konnten die Maßnahmen in den SU maßgeblich unterstützt und notwendige Ressourcen für das Ressort gesichert werden (z. B. Beschaffung von Schutzausrüstung, Erweiterung der Unterbringungs- und Quarantänekapazitäten; Tab. 1).

### Verknüpfung zentraler Informationen mit lokaler Expertise

In den Interviews zeigte sich durchweg ein hoher Bedarf an einer wissenschaftlichen Bewertung verschiedener Maßnahmen sowie praktischer, infektionsepidemiologischer Expertise bei Entscheidungen vor Ort. Alle Teilnehmer*innen nannten die allgemeinen RKI-Richtlinien zu COVID-19 als zentrale Informationsquelle, vor allem zur Orientierung in Sachen physischer Distanzierung, Testung und Quarantänemaßnahmen. Zusätzlich orientierten sie sich an Vorgaben auf Bundes‑, Landes- und Kreisebene.

Mehreren Interviewteilnehmer*innen zufolge ließen sich viele der bestehenden Vorgaben (z. B. zum Arbeitsschutz, zu Quarantäne) jedoch nicht vollständig auf das Setting der SU übertragen. Die baulichen Gegebenheiten sowie Landesvorschriften zur Mindestbelegung, durch welche viele Geflüchtete auf sehr engem Raum untergebracht sind, führten zum Beispiel dazu, dass die effektive Trennung Infizierter von Nichtinfizierten „nur auf dem Papier“ (31EA) ermöglicht werden konnte (Tab. 1).

Neben der allgemeinen Verbesserung der Unterbringungssituation Geflüchteter plädierten die Teilnehmer*innen für konkrete Empfehlungen in Zusammenhang mit der COVID-19-Pandemie. Ein Bedarf für spezifische Empfehlungen wurde dabei besonders im Bereich der konkreten Umsetzung von Infektionsschutz- und Quarantänemaßnahmen in der Unterbringung von Geflüchteten geäußert. Hier wurde vermehrt darauf hingewiesen, dass die Aufnahmebehörden keine medizinische Fachexpertise haben und deshalb für eine Übersicht der neuesten wissenschaftlichen Erkenntnisse und daraus resultierenden Handlungsoptionen auf andere Instanzen angewiesen sind, welche in eine entsprechende bundesweite Strategie eingebunden sind (Tab. 1).

Wegen der sehr unterschiedlichen Bedingungen in jedem Landkreis und auch in jeder Unterkunft wurde zusätzlich zu zentral bereitgestellten wissenschaftlichen Empfehlungen und Handlungsoptionen der Bedarf an lokaler praktischer Expertise und Beistand zur Umsetzung von Empfehlungen vor Ort geäußert. Auch deshalb wurden die Stärkung des ÖGD und dessen stärkere und unmittelbare Einbindung in die Aufgaben der Flüchtlingshilfe als essenziell erachtet (Tab. 1): „[…] die klassische Frage: ‚Gibt es einen Plan für unsere Unterkunft, wenn wir einen Fall haben?‘ die wurde dann oft damit beantwortet mit ‚naja dann machen wir einen Plan‘ – da hätte ich mir gewünscht, dass man sich da auch einfach mal mit dem Gesundheitsamt vielleicht auch mal richtig zusammengesetzt hätte und einen Plan zusammen entwickelt hätte“ (45LK).

## Diskussion

Diese für Deutschland erste empirische Situationsanalyse der ergriffenen Infektionsschutz‑, Präventions- und Informierungsmaßnahmen in Sammelunterkünften für Geflüchtete zeichnet insgesamt ein sehr heterogenes Bild.

Die Situation ist geprägt durch ein hohes Engagement der Akteur*innen vor Ort und verdeutlicht die *zentrale* Rolle von Aufnahmebehörden für die Prävention von COVID-19 und von psychischen Belastungen sowie für die gesundheitliche Aufklärung im Setting SU. So übernahmen sie vielerorts Verantwortung für die Kommunikation mit Geflüchteten, kommunalen Akteur*innen und Landes- bzw. Bundesbehörden. Soweit möglich, stellten sie zur Prävention psychischer und körperlicher Erkrankungen unter Pandemiebedingungen die gesundheitliche Versorgung und soziale Beratung weiterhin sicher. Jedoch sind die Aufnahmebehörden nicht *per se* mit der notwendigen Expertise im Bereich Gesundheitsschutz, Medizin und Public Health ausgestattet, um diesen komplexen Aufgaben nachzukommen. Zudem ist das Selbstverständnis, diese Aufgaben zu übernehmen, unterschiedlich ausgeprägt. Auch unterscheidet sich das Selbstverständnis in Abhängigkeit von den föderalen Ebenen. So kommt im Zuge des Sicherstellungsauftrags der medizinischen Versorgung den Aufnahmebehörden in den EA im Allgemeinen eine größere Rolle zu als z. B. in den GU, wo die Regelversorgung eine größere Rolle spielt [4]. Eine enge Zusammenarbeit zwischen Gesundheitsamt und Aufnahmebehörde war in dieser Studie eine Ausnahme. Selbst wenn eine Erreichbarkeit vorlag und es zu einer Unterstützung der Aufnahmebehörden vor Ort kam, umfasste diese zwar praktische Aspekte der Testung oder Quarantäne, nur in Einzelfällen aber auch präventivkonzeptionelle Aspekte.

Aus den Interviews lassen sich 4 prioritäre Bedarfe im Kontext der Pandemie ableiten:

### Erstens eine allgemeine Verbesserung der Unterbringungssituation für Geflüchtete, inklusive räumlicher Kapazitäten für eine entzerrte Unterbringung und Isolations- und Quarantänemaßnahmen.

Die Relevanz dieser Forderung der Aufnahmebehörden bekommt insbesondere dort eine Dringlichkeit, wo die Umsetzung von präventiven Maßnahmen durch bauliche Bedingungen erschwert wurde oder Quarantänemaßnahmen und die effektive Trennung Infizierter von Nichtinfizierten im Ausbruchsfall nicht umgesetzt werden konnten.

### Zweitens zentrale sowie settingspezifische Vorgaben, die die Situation in den Sammelunterkünften adäquat berücksichtigen und über allgemeine Empfehlungen hinausgehen.

(Anmerkung: Die spezifischen Empfehlungen des RKI zum Umgang mit der COVID-19-Pandemie in Aufnahmeeinrichtungen [10] wurden erst nach Abschluss dieser Studie am 10.07.2020 veröffentlicht.) Empfehlungen und Informationsmaterial – auch von Bundesebene – werden wahrgenommen und genutzt, jedoch besteht ein hoher Bedarf an settingspezifischen Anpassungen, darunter zum Beispiel der Umgang mit Quarantänemaßnahmen, welcher den notwendigen Infektionsschutz mit den psychischen Belastungen langwieriger und erzwungener Isolation abwägt. Diese Vorgaben sollten neben essenziellen Infektionsschutzmaßnahmen auch soziale und psychische Bedarfe berücksichtigen, um zusätzliche Stressoren unter Geflüchteten während der Pandemie zu minimieren.

### Drittens eine enge Kooperation zwischen Aufnahmebehörden und lokaler, gesundheitlicher Fachexpertise, um eine kontextspezifische Implementierung zentraler Vorgaben unter Berücksichtigung der jeweils lokalen Situation zu ermöglichen.

Diese beinhaltet explizit die frühzeitige Planung und Umsetzung präventiver und allgemeiner Infektionsschutzmaßnahmen, aber auch die antizipierende Planung von zu ergreifenden Maßnahmen im Ausbruchsfall.

### Viertens ein umfängliches, zentral gesteuertes und wissenschaftlich begutachtetes Informationsangebot für das Setting der SU unter Berücksichtigung der Notwendigkeit von Mehrsprachigkeit, einfacher Sprache und Piktogrammen für die effektive Kommunikation mit Geflüchteten. 

Dieses sollte flankiert werden von lokalen Strukturen, die einen systematischen Einsatz von geschulten Sprachmittler*innen in der gesundheitlichen Prävention in SU ermöglichen, um über geltende Maßnahmen und Regelungen aufzuklären, Bedenken und Ängste zu adressieren und Akzeptanz herzustellen.

Vergleicht man die Ergebnisse dieser Studie mit bestehenden Erkenntnissen zur gesundheitlichen Versorgung von Geflüchteten, ist zu erkennen, dass die im Zuge der Pandemie getroffenen Maßnahmen die Heterogenität der Strukturen des Flüchtlingskontexts widerspiegeln. Diese sind, sowohl im Sommer der Migration 2015 [4] als auch mehrere Jahre danach, in Fragen des Gesundheitsschutzes [12], der Prävention und Gesundheitsförderung sowie der allgemeinen gesundheitlichen Versorgung [13, 14] durch eine ausgeprägte Diversität von Ansätzen, Vorgehen und Lösungen charakterisiert. In diesem Zusammenhang wurde bereits gezeigt, dass das komplexe Versorgungsgefüge zwischen Behörden, Regelversorgung und ÖGD zu Zugangsbarrieren und Versorgungslücken in der praktischen Gesundheitsfürsorge bei der Gruppe der Geflüchteten führen kann [4, 15]. Weiterhin notwendig ist eine breitere Diskussion über die Rolle und das Selbstverständnis des ÖGD in Zusammenhang mit Prävention, Gesundheitsförderung und medizinischer Versorgung Geflüchteter [4], insbesondere angesichts der Herausforderungen der COVID-19-Pandemie [5]. Hierfür sollten Erfahrungen guter Praxis aus 2015 zu Modellansätzen der Einbindung des ÖGD in die Flüchtlingsversorgung dringend breitere Anwendung finden [14, 16].

Diese Studie profitiert von einem qualitativen Ansatz, welcher tiefe Einblicke in den Kontext und die derzeitige Arbeitsweise der Aufnahmebehörden erlaubte. Aufgrund mehrjähriger qualitativer Forschungserfahrung der Interviewer*innen in diesem Kontext und unter Wahrung der Anonymität von Teilnehmer*innen wurden sensitive Fragen in aller Regel sehr offen beantwortet. Eine weitere Stärke ergibt sich durch die Anzahl der Teilnehmer*innen und die Bandbreite der abgedeckten Bundesländer, wodurch Einblicke in diverse infrastrukturelle und behördliche Strukturen gewonnen werden konnten. Der kurze Zeitraum der Erfassung ermöglichte zudem eine zeitnahe, der Dringlichkeit der Pandemie angemessene Situationsanalyse. Eine Schwäche der Studie auf GU-Ebene ergibt sich durch den starken geografischen Fokus auf ein Bundesland. Jedoch kamen bei GU in anderen Bundesländern keine gänzlich neuen Aspekte hinzu, sodass davon auszugehen ist, dass die Heterogenität innerhalb des Flächenlands die Situation in anderen Flächenländern mit ähnlicher Struktur widerspiegelt. Die Aussagen beziehen sich auf den ersten Abschnitt der SARS-CoV-2-Pandemie in Deutschland (Erhebungszeitraum Mai–Juli 2020). Es ist nicht auszuschließen, dass in Anbetracht der dynamischen Pandemielage eine Folgebefragung neue Kategorien und Dimensionen bezüglich der Fragestellung hervorbringen würde.

## Fazit

Die Heterogenität der im Zuge der COVID-19-Pandemie getroffenen Maßnahmen lässt sich zum Großteil auf die hohe Anzahl an Akteur*innen sowie der Komplexität von Strukturen und Prozessen der Aufnahme und gesundheitlicher Versorgung Geflüchteter zurückführen. Diese waren vorbestehend und zuletzt auch 2015 deutlich zutage getreten. Im Geflecht dieser Strukturen übernehmen Aufnahmebehörden für die Sicherstellung der Gesundheit *ad hoc* essenzielle Aufgaben, für die sie jedoch fachlich und praktisch unzureichend von lokalen, regionalen und bundesweiten Instanzen unterstützt werden. Für eine effektive Eindämmung der COVID-19-Pandemie und weitere gesundheitliche Herausforderungen sind eine gesteuerte und settingspezifische Bündelung fachlicher Empfehlungen und Information auf Bundesebene sowie die stärkere, proaktive Einbindung des öffentlichen Gesundheitsdienstes in die Planung und Umsetzung der Maßnahmen vor Ort unabdingbar.

## Supplementary Information




