# Supplementary material for: COVID-19 in collective accommodation centres for refugees: assessment of pandemic control measures and priorities from the perspective of authorities
Source: Bundesgesundheitsblatt Gesundheitsforschung Gesundheitsschutz. 2021 Feb 9;64(3):342–52. [Article in German] doi: 10.1007/s00103-021-03284-2 (PMC7872106; doi:10.1007/s00103-021-03284-2)
Supplement: Supplementary file 1 [file 103_2021_3284_MOESM1_ESM.pdf]

Bundesgesundheitsblatt 3/2021

Elektronisches Zusatzmaterial zum Beitrag:

COVID-19 in Sammelunterkünften für Geflüchtete: Analyse von Pandemiemaßnahmen und prioritäre Bedarfe aus behördlicher Sicht

Louise Biddle[1], Rosa Jahn[1], Clara Perplies[1], Andreas W. Gold[1], Eilin Rast[1], Anke Spura[2], Kayvan Bozorgmehr[1,3]

[1] Sektion Health Equity Studies und Migration, Abteilung für Allgemeinmedizin und Versorgungsforschung, Universitätsklinikum Heidelberg, Heidelberg, Deutschland
[2] Bundeszentrale für Gesundheitliche Aufklärung, Köln, Deutschland
[3] Abteilung Bevölkerungsmedizin und Versorgungsforschung, Fakultät für Gesundheitswissenschaften, Universität Bielefeld, Bielefeld, Deutschland

Korrespondenzadresse:

Prof. Dr. Kayvan Bozorgmehr (MSc)
Sektion Health Equity Studies und Migration
Abteilung Allgemeinmedizin und Versorgungsforschung
Im Neuenheimer Feld 130.3
69120 Heidelberg
kayvan.bozorgmehr@med.uni-heidelberg.de

Inhalte:

1. **Semi-strukturierter Leitfaden**
2. **Codesystem zur Auswertung der Interviews**

1. Semi-strukturierter Leitfaden

1. Können Sie uns bitte kurz erläutern, wie Sie mit dem neuartigen Coronavirus in Ihren Aufnahmeeinrichtungen/ Gemeinschaftunterkünften umgehen?
2. Gibt es landesspezifische/ landkreisspezifische Vorgaben zum Umgang und wenn ja welche?
3. Über welche Quellen beziehen Sie Ihre Informationen zum Umgang mit der Situation?
4. Wie erfahren Sie, dass es in Ihrer Unterkunft einen (potenziellen) COVID-19-Fall gibt? Was passiert dann?
5. Wie werden die Maßnahmen sozialer Distanzierung in den Unterkünften umgesetzt?
6. Wie erfolgen denn derzeit Angebote, die normalerweise in den Unterkünften gewährleistet werden, wie zum Beispiel die der Verfahrens- und Sozialberatung?
7. Wie informieren Sie die Bewohner über die Maßnahmen?
8. Mit welchen Akteuren auf Landes-/Landkreisebene arbeiten Sie im Zusammenhang mit der Versorgung Geflüchteteter eng zusammen? Wie ist die Zusammenarbeit organisiert?
9. Wir haben jetzt verschiedene Punkte rund um den Umgang mit COVID-19 angesprochen. Gibt es aus Ihrer Sicht noch wichtige Punkte, die wir bisher noch nicht besprochen haben?

2. Codesystem zur Auswertung der Interviews

1. Informieren von Bewohner*innen
    a. Art der Information
    b. Inhalt
    c. Übersetzung & Sprachmittlung
    d. Quellen Infomaterial
    e. Eigeninformation der Bewohner*innen
    f. Unterstützungsbedarf Informieren
    g. Umgang von. Bewohner*innen mit Informationen
    h. Sonstiges
2. Physische Distanzierung Bewohner
    a. Entzerrung der Belegung
    b. Hinderungsgründe u. Herausforderungen Entzerrung Belegung
    c. Besuchs- u. Betretungsverbote
    d. Anpassungen Kochsituation u. Essensausgabe
    e. Abstandsregelungen Bewohner*innen miteinander
    f. Separierung Risikogruppen
    g. Umgang Bewohner*innen mit physischer Distanzierung
    h. Vorgehen Regelverstoß
    i. Vorgaben und Guidelines zu Distanzierung
    j. Unterstützungsbedarf Distanzierung
    k. Sonstiges
3. Schutz- & Hygienemaßnahmen
    a. MNS (Mund-Nase-Schutz)
    b. Bereitstellung Desinfektions- und Hygieneprodukte
    c. Anpassungen im Reinigungsprozess
    d. Beschaffung von Materialien (Bewohner*innen & Mitarbeiter*innen (MA))
    e. Unterstützungsbedarf bei Schutz-und Hygienemaßnahmen
    f. Vorgaben und Guidelines zu Schutz- & Hygienemaßnahmen
    g. Sonstiges
4. Physische Distanzierung Mitarbeitende
    a. Vermeidung physischer Kontakte
    b. Schutzausrüstung
    c. Abstandregelungen
    d. Spuckschutz
    e. Schichtdienst & Terminplanung
    f. Arbeitsbelastung der MA
    g. Umgang und Einstellungen der MA
    h. Vorgaben und Guidelines
    i. Unterstützungsbedarf Distanzierung MA
    j. Sonstiges
5. Angebote
    a. Sozial-/Verfahrensberatung
    b. Freizeit-, Bildungs-, Beschäftigungsangebote
    c. ehrenamtliche Angebote
    d. Sonstiges
    e. Vorgaben/Guidelines zu Angebotsanpassungen
6. Intersektorale Zusammenarbeit
    a. Allgemein
    b. Gesundheitsamt
    c. Weitere Akteure

    d. Föderalismus
    e. Unterstützungsbedarf intersektorale Zusammenarbeit
    f. Krisenstab/Taskforce
    g. Informationsquellen
    h. Sonstiges
7. Gesundheitsversorgung
    a. zusätzlicher Versorgungsbedarf
    b. angepasste & neue GesV-Strukturen
    c. vor Ort
    d. Extern
    e. Behandlungsscheine
    f. Quarantäne
    g. Vorgaben/Guidelines zu Gesundheitsversorgung
    h. Sonstiges
8. Testung
    a. Informationswege Testergebnisse
    b. Mitteilung Testergebnisse an Bewohner*innen
    c. Testungsanlass
    d. Test-Isolierung
    e. Testung von Mitarbeitenden
    f. Vorgaben/Guidelines zu Testung
    g. Unterstützungsbedarf bei Testung
    h. Sonstiges
9. Eindämmungsmaßnahmen
    a. Quarantäne
    b. Isolierung
    c. Kapazitäten
    d. Erwartete Maßnahmen
    e. Umgang der Bewohner*innen mit Maßnahmen
    f. Angebote in Quarantäne & Isolierung
    g. Vorgaben/Guidelines zu Eindämmungsmaßnahmen
    h. Unterstützungsbedarf bei Eindämmungsmaßnahmen
    i. Sonstiges
10. Politischer Handlungsspielraum
    a. Zuständigkeiten & Vorgaben
    b. Einschränkungen durch fehlende Befugnisse
    c. Handlungsspielraum von lokalen Strukturen abhängig
    d. Flucht auf lokalpolitischer Agenda
    e. Führung/ Governance
    f. Sonstiges
11. Setting
    a. Corona-Fälle in Unterkunft und Kreis
    b. Größe & Anzahl der Unterkunft und Größe des Kreises
    c. Einrichtungsstruktur
    d. Motivation zur Auseinandersetzung mit COVID-19
    e. Sonstiges
12. Sonstiges allgemein
